# *Kirrel3 is associated with disordered sleep in presymptomatic Alzheimer’s disease mice*.

**DOI:** 10.1002/alz.095499

**Published:** 2025-01-09

**Authors:** Surjeet Singh, Niran Hadad, Amy R Dunn, Harpreet Kaur, Julia A. Kravchenko, Kristen O'Connell, Timothy J. Hohman, Allan I Pack, Catherine C. Kaczorowski

**Affiliations:** ^1^ The Jackson Laboratory, Bar Harbor, ME USA; ^2^ University of Michigan, Ann Arbor, MI USA; ^3^ The Translational Genomics Research Institute, Phoenix, AZ USA; ^4^ Vanderbilt Memory and Alzheimer’s Center, Institute for Medicine and Public Health, Vanderbilt University Medical Center, Nashville, TN USA; ^5^ Vanderbilt Genetics Institute, Institute for Medicine and Public Health Vanderbilt University Medical Center, Nashville, TN USA; ^6^ Perelman School of Medicine at the University of Pennsylvania, Philadelphia, PA USA

## Abstract

**Background:**

Sleep dysfunctions are highly comorbid with Alzheimer’s disease (AD), though often associated with later stages of AD, sleep disruptions have been noted to appear decades before the onset of cognitive symptoms. Here, we provide the first evidence that genetic factors interact with AD mutations to influence sleep behavior even before the onset of cognitive symptoms.

**Method:**

To identify novel genetic factors underlying disordered sleep that precede cognitive decline in our AD‐BXD mouse genetic reference panel (n = 179 mice across 25 strains, 7‐months‐old), we first used sleep phenotypes measured in the PiezoSleep chambers and performed quantitative trait loci (QTL) mapping and discovered *Kirrel3* as the novel gene candidate associated with disordered sleep. To further evaluate the causal role of *Kirrel3* in susceptibility to sleep decline in the presence of Alzheimer’s disease pathology, we generated mice heterozygous for the *Kirrel3* gene with and without the 5XFAD transgene, and then did sleep phenotyping in these mice (n = 153, 7‐months‐old).

**Result:**

We found that in AD‐BXD mouse population sleep was highly heritable (*h* ^2^ *_RIx̄_ =* 22% – 71%) and varied by light/dark cycle, sex, and the presence of the 5XFAD transgene. This implies high heritability of sleep that lays within the heritability observed in humans. Using QTL mapping we then identified *Kirrel3* as a novel genetic factor underlying disordered sleep that precedes AD‐related cognitive decline in mice. Further evaluation of sleep changes in *Kirrel3* heterozygous mice revealed a significant reduction in total sleep and sleep during dark cycle, for both sexes but only in the presence of 5XFAD transgene.

**Conclusion:**

Recent GWAS studies in humans nominated Kirrel3 (aka Neph2) as a potential AD risk gene and as a regulator of sleep behavior (independent of disease). In this study we identified *Kirrel3* as a novel genetic factor contributing to disordered sleep that precedes cognitive decline in our AD‐BXD mouse genetic reference panel. Further, using Kirrel3 heterozygous mice, we provide, first evidence for the causal role of *Kirrel3* in sleep regulation only in the presence of 5XFAD transgene.

